# CD8^+^ Resident Memory T Cells and Viral Infection

**DOI:** 10.3389/fimmu.2018.02093

**Published:** 2018-09-19

**Authors:** Xuejie Wu, Pin Wu, Yifei Shen, Xiaodong Jiang, Feng Xu

**Affiliations:** ^1^Department of Infectious Diseases, The Second Affiliated Hospital, Zhejiang University School of Medicine, Hangzhou, China; ^2^Department of Thoracic Surgery, The Second Affiliated Hospital, Zhejiang University School of Medicine, Hangzhou, China; ^3^Department of Immunobiology, Yale University School of Medicine, New Haven, CT, United States

**Keywords:** memory T cells, tissue-resident memory T cells, microenvironment, viral infection, immune response

## Abstract

Tissue-resident memory T (Trm) cells are a subset of recently identified memory T cells that mainly reside and serve as sentinels in non-lymphoid peripheral tissues. Unlike the well-characterized circulating central memory T (Tcm) cells and effector memory T (Tem) cells, Trm cells persist in the tissues, do not recirculate into blood, and offer immediate protection against pathogens upon reinfection. In this review, we focus on CD8^+^ Trm cells and briefly introduce their characteristics, development, maintenance, and function during viral infection. We also discuss some unresolved problems, such as how CD8^+^ Trm cells adapt to the local tissue microenvironment, how Trm cells interact with other immune cells during their development and maintenance, and the mechanisms by which CD8^+^ Trm cells confer immune protection. We believe that a better understanding of these problems is of great clinical and therapeutic value and may contribute to more effective vaccination and treatments against viral infection.

## Introduction

Upon infection, the host immune system initiates immune responses against invading pathogens, a process in which both innate and adaptive immune cells participate sequentially and synergistically. Pathogen-specific memory T cells and B cells persist long after the infection has been cleared (1–3). Until recently, memory T cells had been categorized into central memory T (Tcm) cells and effector memory T (Tem) cells. Tcm cells are a small population of memory T cells that circulate between the secondary lymphoid organs (SLOs) and the blood. They are long-lived and can be activated rapidly upon reencountering their cognate antigen in SLOs. Tem cells have been proposed to migrate through the blood, lymphoid and non-lymphoid tissues (NLT). They kill pathogens via a variety of effector mechanisms and disappear gradually after the pathogens have been eliminated (4–8).

Resident memory T (Trm) cells, a third population of memory T cells, have been identified recently, especially in barrier tissues and SLOs (6, 9–12). They persist permanently in these tissues and do not recirculate into the blood. They can mount a rapid immune response upon reencountering the same pathogen and restrict infection within the local tissue sites (11–14). In addition, emerging data indicate that Trm cells are also involved in tumor immunosurveillance (15). CD103 and CD69 have been considered as two common surface markers in distinguishing Trm cells from other memory T cells (16–20). However, some studies have demonstrated that CD69^−^ or CD103^−^ Trm cells also exist in non-lymphoid peripheral tissues (21–23). This indicates that the development and maintenance of Trm cells, including their phenotypic characteristics, are tightly regulated by local microenvironment (24, 25).

While both CD4^+^ and CD8^+^ Trm cells have been identified, CD8^+^ Trm cells are more extensively investigated in viral infection (26). CD8^+^ Trm cells that arise through infections due to a variety of pathogens have been identified and characterized in many studies. For example, after acute herpes simplex virus (HSV) infection, CD8^+^ Trm cells are generated and retained in the skin to protect against reinfection of HSV (27). Skin CD8^+^ Trm cells produce abundant interferon (IFN)-γ and tumor necrosis factor (TNF)-α following cognate antigen stimulation and are responsible for efficient control of vaccinia virus (VACV) re-infection (12). Using intraglandular infection, Thom et al. demonstrated that CD8^+^ Trm cells immediately defend the host against local murine cytomegalovirus (MCMV) infection, despite active viral immune evasion (28). Influenza virus-specific CD8^+^ Trm cells in the nasal epithelia prevent the transmission of influenza virus from the upper respiratory tract to the lung (29). These cells are also sequestered in the walls of the large airways and are crucial for ideal cross-protection against pulmonary influenza virus infection (30–32). Intranasal vaccination of live-attenuated influenza virus generates virus-specific CD8^+^ Trm cells as well (33). Moreover, both mouse and human respiratory syncytial virus (RSV) specific CD8^+^ Trm cells are associated with control of lung RSV infection (34, 35). Immune responses of human immunodeficiency virus (HIV)-1-specific CD8^+^ Trm cells are the strongest in patients whose immune systems are able to naturally control HIV-1 infection, suggesting the involvement of these cells in local anti-HIV immunity (36). In immunosuppressed renal transplant recipients (RTRs), impaired effector differentiation of polyomavirus BK (BKPyV) major capsid protein (VP1)-specific CD8^+^ Trm cells is associated with BKPyV-induced interstitial nephritis (BKVN), which is caused by BKPyV reactivation after initial control of the virus (37). In addition to non-lymphoid peripheral tissues, CD8^+^ Trm cells are also embedded in thymus and mediate local immunity against lymphocytic choriomeningitis virus (LCMV) reinfection through degranulation and cytokine (IFN-γ and TNF-α) production (38). Together, all these data indicate that CD8^+^ Trm cells play an important role in anti-viral immunity not only in non-lymphoid peripheral tissues but also in lymphoid tissues.

In this review, we mainly focus on CD8^+^ Trm cells and briefly introduce their characteristics, development, maintenance and functions in viral infection. We also discuss the impact of local tissue microenvironment on determining phenotypes of CD8^+^ Trm cells, the mutual conversion of Trm, Tem, and Tcm cells, the mechanisms of long-term maintenance of Trm cells, and crucial steps in initiating CD8^+^ Trm cell immune responses. To understand these fundamental questions and further illustrate the underlying mechanisms will help find better strategies for control of viral infection.

## Characteristics of CD8^+^ resident memory T cells

Unlike circulating CD8^+^ Tcm and Tem cells, CD8^+^ Trm cells locate permanently in the tissues and do not recirculate into the blood (39). More importantly, CD8^+^ Trm cells are distributed widely in non-lymphoid peripheral tissues including the skin, lung, gastrointestinal tract, female reproductive tract (FRT), brain, liver, kidney, salivary glands, etc (16, 18, 40–44). Recently, some studies reported that CD8^+^ Trm cells also persist in lymphoid tissues including SLOs and thymus (11, 38, 45). The broad distribution of Trm cells indicates their importance in local immunity.

CD8^+^ Trm cells in non-lymphoid tissues were initially defined as CD103^+^ CD69^+^ (16–20). But later CD69^−^ and CD103^−^ Trm cells were also identified, suggesting that CD69 and CD103 may not be the definite markers of Trm cells (21–23). Interestingly, to some extent these CD103 or CD69 negative CD8^+^ Trm cells are different from those positive populations. For example, both CD69^+^ and CD69^−^ CD8^+^ Trm cells were identified in the pancreas, salivary gland (SG) and FRT, but they have different population sizes (21). In *Yersinia pseudotuberculosis* (Yptb) oral infection model, CD103^+^ CD8^+^ Trm cells are mainly localized in the intestinal epithelium (IEL) and lamina propria (LP) while CD103^−^ CD8^+^ Trm cells mainly reside in LP and are close to the crypts (46). CD103^+^ CD8^+^ and CD103^−^ CD8^+^ Trm cells are found preferentially in epidermis and in dermis, respectively (18). After murine polyomavirus (MuPyV) infection, brain CD103^+^ CD8^+^ Trm cells uniformly express programmed cell death protein 1 (PD-1), in contrast to CD103^+^ CD8^+^ Trm cells in the spleen, which are PD-1 negative (23). In addition, CD8^+^ Trm cells within intestinal mucosa express a variety of distinct molecules that distinguish themselves from memory T cells in SLOs: up-regulate CD28 and CD11c and rapidly produce IFN-γ after reactivation by antigen (47).

Like circulating Tcm and Tem cells, CD8^+^ Trm cells in different tissues also have distinct transcriptional programs. Lung, skin or gut CD8^+^ Trm cells have a unique core transcriptional profile with 25–127 specific transcripts, which are progressively engaged during differentiation (18). Liver, known as an immune tolerance organ, retains large numbers of CD8^+^ Trm cells that express low levels of sphingosine 1-phosphate receptor-1 (S1PR1) and Krüppel-like Factor 2 (KLF2); interestingly, most of these CD8^+^ Trm cells in the liver are CXCR6 and granzyme positive, and are localized in portal fields, central veins, and parenchymal zones in CHB patients (48). CD8^+^ Trm cells isolated from the brain possess altered molecular signatures including chemokines and chemokine receptors (up-regulation of CCL3, CXCL10, and CCL4 and down-regulation of CX3CR1 and CCL9), transcription factors (down-regulation of eomes, Tcf-1, lef1, and T-bet and up-regulation of IFITM3, Irf4, and Isg20) and several inhibitory receptors (CTLA-4 and PD-1) after recombinant vesicular stomatitis virus (VSV) infection (49). Similar to mouse CD8^+^ Trm cells, human CD8^+^ Trm cells up-regulate ITGA1 (CD49a), ICOS, and the transcription factor IRF4 but down-regulate eomes (43, 50).

CD8^+^ Trm cells can mount a rapid and robust immune response against reinfection, which is thought to be critical for the efficacy of vaccination. Some functional differences between Trm populations among children, adults, and the elderly have been observed (51). Compared to adults, fewer lung CD8^+^ and CD4^+^ Trm cells are established after influenza infection during infancy, which may be associated with more serious or frequent respiratory infections and reduced vaccine responses. The difference between adult and infant Trm cell establishment can be attributed to increased T-bet expression in infant T cells after activation, as is demonstrated in both murine and human models (52).

Taken together, current studies indicate that CD8^+^ Trm cells in different tissues share some common characteristics in phenotype and functions. However, they also have distinct properties in phenotypes, transcriptional profiling and function as well. The differences among them may be caused by the regulation of their unique tissue microenvironment, which affects their developmental fates.

## Development of CD8^+^ resident memory T cells

How memory T cells are generated is a fundamental question in the research field of immunological memory. For classical Tcm and Tem cell development, there are several differentiation hypotheses including linear differentiation model and asymmetric division model (53–55). CD127^+^ killer cell lectin-like receptor G1 (KLRG1)^−^ CD8^+^ T cells have been demonstrated to be memory precursor effector cells (MPECs) (56). Whether CD8^+^ Trm cells also have precursors and what the underlying transcriptional mechanisms in CD8^+^ Trm cell development are critical questions in the research field of Trm cells.

Mackay et al. (18) recently found that KLRG1^−^, not KLRG1^+^, activated CD8^+^ T cells can develop into skin epithelium-infiltrating CD103^+^ CD8^+^ Trm cells. CD127^+^ KLRG1^−^ CD8^+^ T cells have been demonstrated to be the intestinal CD8^+^ Trm precursors in an oral *Listeria monocytogenes* infection model (57). However, CD127^+^ KLRG1^+^ effector CD8^+^ T cells may lose KLRG1 and differentiate into all memory T cell lineages including CX3CR1^−^ Trm cells (58, 59). Gerlach et al. recently demonstrated that CX3CR1 is a critical chemokine receptor correlated with CD8^+^ T cell differentiation and further suggested that CD8^+^ Trm cells are derived from CX3CR1^−^ activated CD8^+^ T cells (59). It was reported that DC NK lectin group receptor-1 (DNGR-1)^+^ dendritic cells (DCs) may prime naïve CD8^+^ T cells to become Trm cell precursors in draining lymph nodes (dLNs), but are not required for Trm differentiation in the skin. Expression of interleukin (IL)-12, IL-15, and CD24 is essential for optimal formation of Trm cells (60). To date, how DC subsets play an important role in generating CD8^+^ Trm cell precursor is still unclear. In addition, it is known that CD4^+^ T cell help is required for DCs to induce a robust effector CD8^+^ T cell response (61). In the absence of CD4^+^ T cells, fewer CD103^+^ CD8^+^ Trm cells are developed in the lungs. Reduced expression of CD103 results from increased expression of the transcription factor T-bet in “unhelped” lung Trm cells. Generation of CD103^+^ CD8^+^ Trm cells also requires CD4^+^ T cell-derived IFN-γ (62). However, in acute VACV skin infection mouse model we did not see a reduction of skin CD8^+^ Trm cells in the absence of CD4^+^ T cells, though the function of skin CD8^+^ Trm cells was found to be partially impaired (12). Moreover, we also did not see any significant reduction of CD8^+^ Trm cells in the absence of IFNγ (our unpublished data).

Several distinct transcription factors or proteins are involved in the development and homeostasis of CD8^+^ Trm cells. For instance, CD8^+^ Trm cells can utilize the transcription factor AhR to maintain residency in the epidermis and compete with dendritic epidermal γδ T cells for space within the epidermal niche (63). In mice, development of CD8^+^ Trm cells in the skin, gut, liver, and kidney requires cooperation of transcription factors Hobit and Blimp1 (64). Moreover, the function and development of Trm cells can be influenced by nuclear receptor subfamily 4 group A member 1 (NR4A1) and ATP-binding cassette (ABC) transporters (65). Using computational and pooled *in vivo* RNA interference screens, Milner et al. showed that the transcription factor Runx3 also plays a crucial role in the differentiation and homeostasis of CD8^+^ Trm cells (66). Purinergic receptor P2RX7 has recently been found to be involved in the generation of CD8^+^ Trm cells in various non-lymphoid sites (67). In addition to local antigen presentation, intrinsic 4-1BB signals are essential in mediating the generation of CD8^+^ Trm cells in the lung during influenza infection (31, 68, 69).

Furthermore, peripheral tissue microenvironment is crucial in shaping the development of CD8^+^ Trm cells. Hair follicle derived cytokines such as IL-7 and IL-15 play critical roles in skin Trm cell homeostasis (70), while transforming growth factor (TGF)-β promotes the formation of kidney CD8^+^ Trm cells by enhancing expression of E- and P-selectin and chemokine receptor CXCR3, which mediate the extravasation of effector T cells (71). Despite the involvement of TGF-β in Trm development, Smad4, which is required for normal differentiation of circulating memory T cells, is not necessary for Trm cell differentiation (72). Adhesion- and degranulation-promoting adapter protein (ADAP) integrin facilitates CD8^+^ Trm cells formation in non-lymphoid tissues (73). Formalin-inactivated RSV combined with CpG (an agonist of TLR9) and L685,458 (an inhibitor of Notch signaling) promote protective CD8^+^ Trm cells in the lungs (74). Additionally, brain TGF-β producing regulatory T cells (Tregs) are found to be involved in CD8^+^ Trm cell accumulation and granzyme B production after West Nile virus (WNV) and MCMV infection (75, 76). Besides non-specific stimulation, specific stimulation such as local antigen in skin is also required for the formation of functional CD8^+^ Trm cells and amplifies their generation. Although recruitment of activated CD8^+^ T cells to VACV infected skin is antigen independent, significant increase in Trm formation is observed when local antigen is present (77). In skin that has been previously infected, antigen-dependent cross-competition is involved in shaping the repertoire of polyclonal antiviral Trm cells (78). Secondary Trm cells form from both pre-existing Trm cells and Trm precursors recruited from the blood in response to local antigen presence (79). Transient introduction of antigen results in the generation of Trm in the brain via an intracranial dendritic cell immunization regimen (80). However, local inflammation in the skin and mucosa alone can drive recruitment of effector populations and direct their conversion to CD8^+^ Trm cells (24). Similarly, differentiation and maintenance of CD8^+^ Trm cells are antigen-independent in small intestine, kidney, pancreas, stomach, heart, and FRT of mice (81, 82).

**Figure 1:**
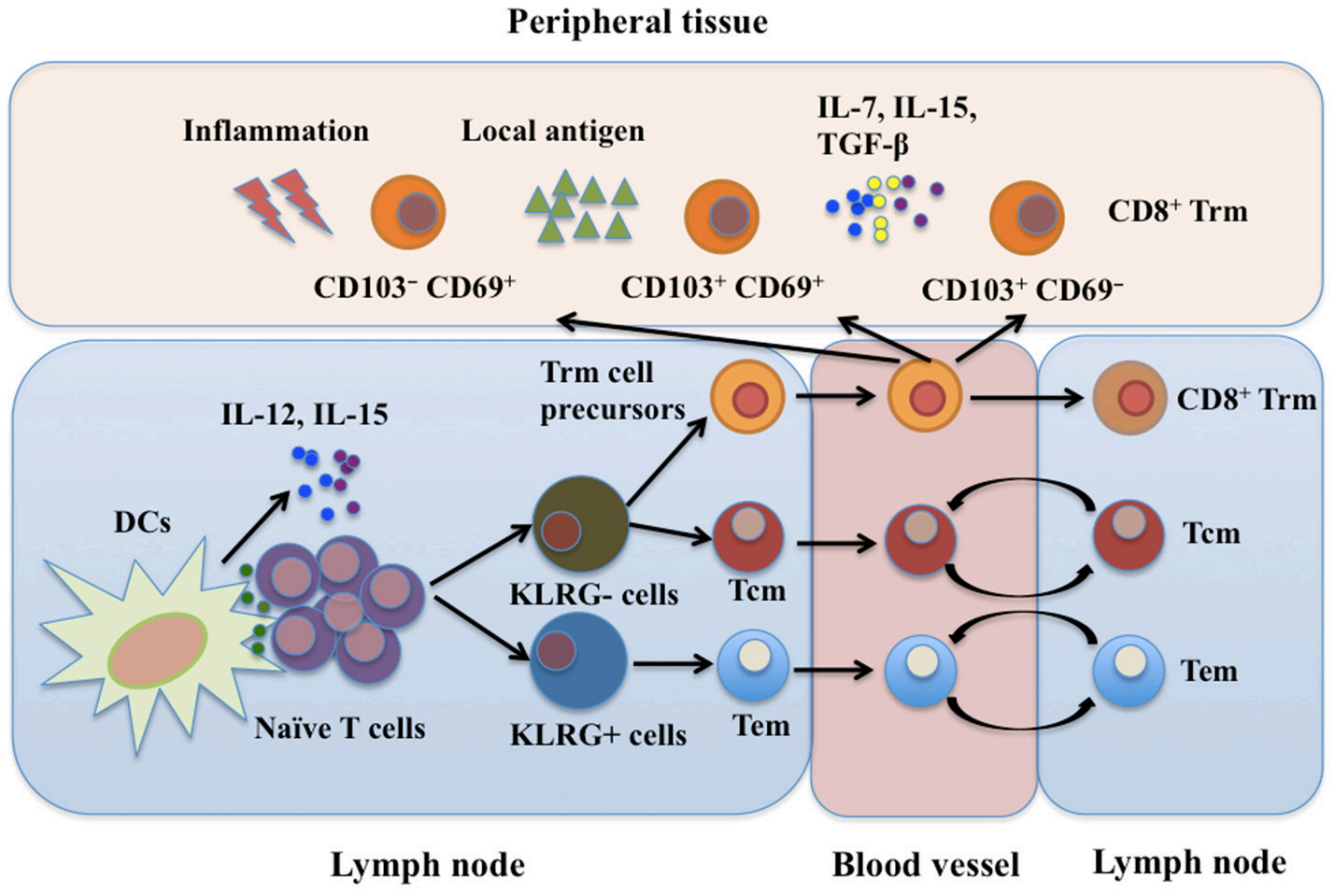
CD8^+^ Trm cell development. Trm, Tem, and Tcm cells are derived from the same naïve T cell clone upon activation in SLOs. CD8^+^ Trm cell precursors migrate into peripheral tissues as well as SLOs where they differentiate into Trm cells. Currently, at least three subtypes of CD8^+^ Trm cells have been identified: CD103^+^ CD69^+^, CD103^+^ CD69^−^, and CD103^−^ CD69^+^ Trm cells. Local microenvironment, including cytokines, local antigens and inflammatory mediators, is important for development of CD8^+^ Trm cells. SLOs, secondary lymphoid organs; Tcm, central memory T cells; Tem, effector memory T cells; Trm, resident memory T cells; DCs, dendritic cells; LN, lymph node; IL-7, interleukin-7; IL-12, interleukin-12; IL-15, interleukin-15; TGF-β, transforming growth factor-β; KLRG, killer cell lectin-like receptor G.

CD8^+^ Trm cells have long been thought to reside exclusively in non-lymphoid tissues. However, in SLOs such as the splenic marginal zone, red pulp, and lymph node sinuses, CD8^+^ Trm cells are also present. These Trm cells can be derived from the skin or mucosa after restimulation (11, 45). Although great progress has been made in characterizing CD8^+^ Trm cell development, the exact mechanisms are still unclear. Both intrinsic and extrinsic factors are involved in the development of CD8^+^ Trm cells. More details need to be known before therapeutically manipulating CD8^+^ Trm cell development, which is important for the control of viral infection and vaccine design. A developmental scheme for CD8^+^ Trm cells is shown in Figure 1.

## Maintenance of CD8^+^ resident memory T cells in local microenvironment

IL-7 and IL-15 are two critical cytokines required for the survival and homeostasis of classical memory CD8^+^ T cells (17, 83). In some but not all peripheral tissues, IL-15 is required for the survival of CD8^+^ Trm cells (18, 84). This suggests that other factors may also be involved in the maintenance of Trm cells. TGF-β is not only critical for Trm formation but also required for skin and gut Trm maintenance (18, 25). Expression of TGF-β and IL-15 are controlled by T-box transcription factors (TFs) Eomes and T-bet (85). Retention of intestinal CD8^+^ Trm cells is partly associated with integrins αEβ7 and α1 as well as CD69, whose expression is induced by TGF-β (25). CD69 may retain Trm cells in the skin by blocking sphingosine-1-phosphate (S1P)-regulated tissue egress (86). Downregulation of S1PR1 is controlled by the transcription factor Kruppel-like factor 2 (KLF2) (87). However, CD69 is not required for Trm cell retention in the lung when they have entered the Trm cell niches (88). In addition, E-cadherin and integrin α4β1 promote CD8^+^ Trm cells accumulation in salivary glands (89, 90).

**Figure 2:**
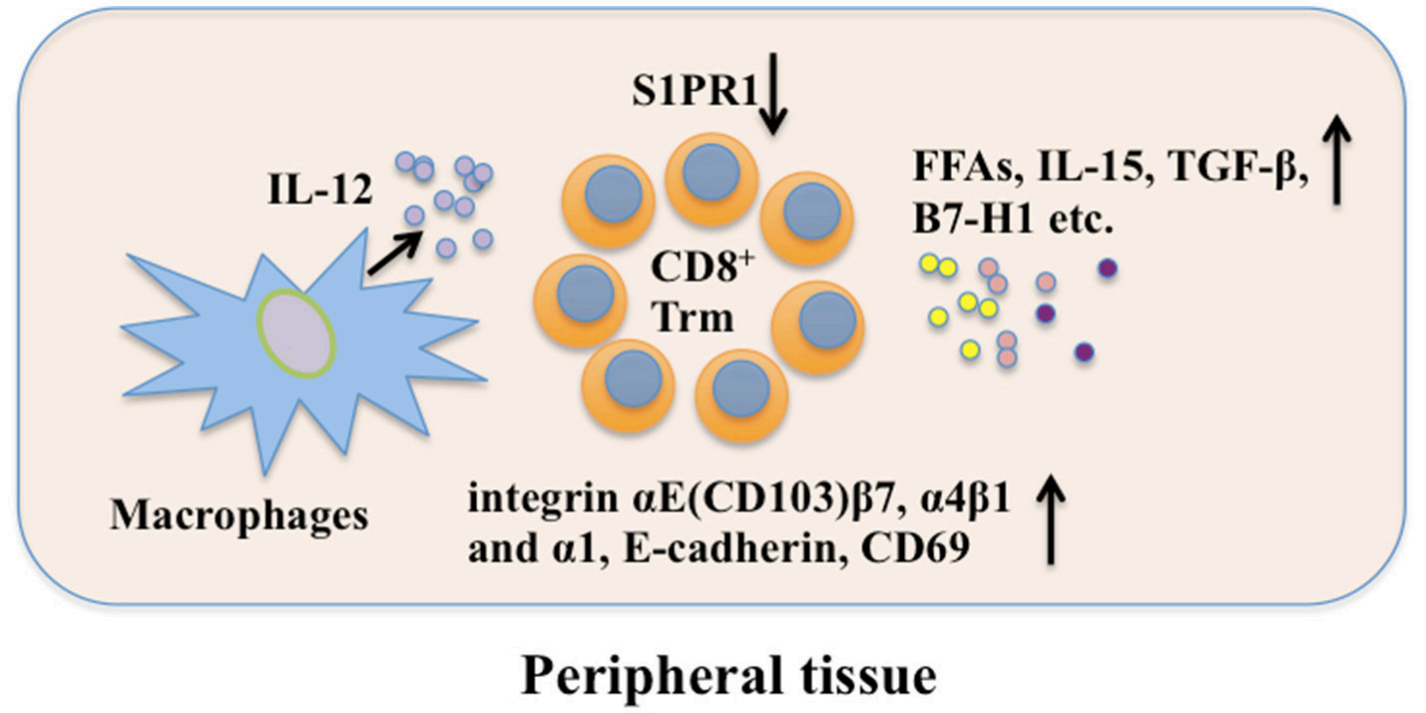
CD8^+^ Trm cell maintenance. Local factors in peripheral tissue are crucial in long-term maintaining CD8^+^ Trm cells. CD69, E-cadherin, and integrin promote retention of CD8^+^ Trm while S1PR1 can mediate CD8^+^ Trm cell tissue egress. FFAs, IL-15, TGF-β, and B7-H1 are involved in CD8^+^ Trm cell maintenance. Besides, macrophages may promote CD8^+^ Trm cell maintenance through secreting IL-12. FFAs, free fatty acids; S1PR1, sphingosine 1-phosphate receptor-1; IL-12, interleukin-12; IL-15, interleukin-15; TGF-β, transforming growth factor-β; B7-H1, B7 homolog 1; Trm, resident memory T cells.

Some other factors also play important roles in maintenance of Trm cells. For example, exogenous free fatty acid (FFA) can be used by skin CD8^+^ Trm cells via fatty-acid-binding proteins (FABP4) 4 and FABP5 for their maintenance (91). Deletion of CCR2^+^ IL-12-producing cells, most of which are macrophages, reduces the size of the CD103^−^ CD8^+^ Trm population during infection, suggesting that macrophages or the mediators they produce may be involved in Trm cell persistence (22). Although local antigen contributes to *in situ* proliferation of Trm cells (77, 78), it is not indispensable for local Trm cell persistence, indicating a dependence on the local microenvironment for Trm function and survival (80). Moreover, CD8^+^ Trm and lymph node Tcm cell clones are generated from the same naïve T cell precursor after skin immunization (92). Therefore, CD8^+^ Tcm could be a potential reservoir for CD8^+^ Trm cells upon reinfection. In addition, reactivation of Trm cells recruit recirculating memory T cells that undergo antigen-independent Trm cell differentiation *in situ* (82). In brain, B7-H1 has a critical role in the maintenance of CD8^+^ Trm cells (93). Understanding how Trm cells maintain long-term residency within barrier tissues will enable the manipulation of these cells *in vitro*. A scheme for possible mechanisms of CD8^+^ Trm cell maintenance is shown in Figure 2.

## Protective mechanisms of CD8^+^ resident memory T cells in antiviral immunity

The importance of CD8^+^ Trm cells in peripheral tissue protection has been widely recognized. Skin CD8^+^ Trm cells serve as sentinels and continuously migrate through the epidermis. They change size, length, and direction of dendrites, which are independent of skin inflammatory state. They quickly identify antigen-expressing cells *in vivo* and initiate *in situ* immune responses (94). The CXCL17/CXCR8 and CXCL10/CXCR3 chemokine pathways are involved in CD8^+^ Trm cell mobilization to infected barrier tissues (95, 96). The rapid control of viral infection is related to abundant IFN-γ and TNF-α produced by CD8^+^ Trm cells following cognate antigen stimulation (12, 97–100). After LCMV reinfection in mice, brain CD8^+^ Trm cells rapidly produce IFN-γ and perforin and prevent fatal brain infection in a manner independent of circulating CD8^+^ memory T cells. Presentation of cognate antigen on major histocompatibility complex (MHC)-I is required for brain Trm cell protective immunity (100). Control of the female mice genital HSV-2 infection by CD8^+^ Trm cells requires expression of MHC-I on CD301b^+^ DCs in the lamina propria (99). However, infected epidermal cells may directly present viral antigen to CD8^+^ T cells to induce cytokine production, which may also be involved in the activation of CD8^+^ Trm cells (94, 101). It is well-known that activated T cells express inhibitory molecules (102). For example, activated intrahepatic CD8^+^ Trm cells express both PD-1 and CD39 after sequential IL-15 or antigen exposure. These inhibitory molecules combined with IL-2 and IFN-γ promote liver CD8^+^ Trm cell survival while contribute to local non-cytolytic hepatic immunosurveillance (103).

**Figure 3:**
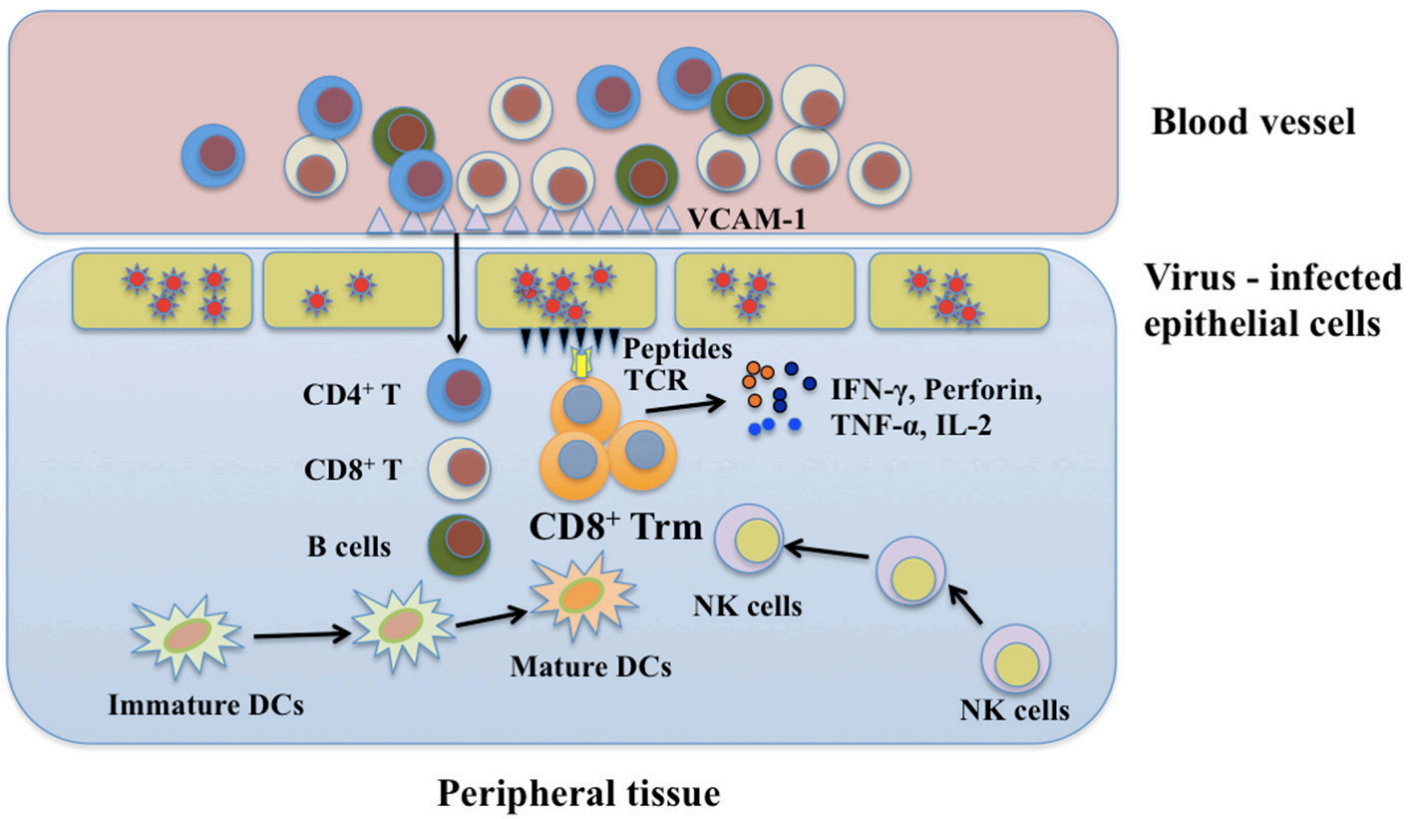
The protective function of CD8^+^ Trm cells in viral infection. Upon encountering the same pathogen, CD8^+^ Trm cells can be reactivated immediately and secrete cytokines in which IFN-γ may help recruit immune cells from blood stream via enhancing expression of endothelial vessel addressin. These immune cells include CD4^+^ T cells, CD8^+^ T cells, and B cells. Besides, NK cells and immature DCs in local tissue can also be recruited to the place where CD8^+^ Trm cells are reactivated. CD8^+^ Trm cells cooperate with these immune cells to synergistically combat with viruses by secreting perforin, IFN-γ, and TNF-α. Trm, resident memory T cells; DCs, dendritic cells; NK, natural killer; IFN-γ, interferon-γ; TNF-α, tumor necrosis factor-α; IL-2, interleukin-2; VCAM-1, vascular cell adhesion molecule-1.

Reactivation of CD8^+^ Trm cells by peptide challenge can trigger strong antiviral immunity against antigenically unrelated pathogens. In addition to inducing a number of broadly active antiviral and antibacterial genes, reactivated Trm cells orchestrate both innate and adaptive immune components including recruitment of recirculating CD4^+^ T cells, CD8^+^ T cells and B cells, maturation of DCs, and activation of natural killer (NK) cells to develop a “pathogen alert” state. Achievement of these functions relies on IFN-γ, TNF-α, and IL-2Rβ-dependent cytokines (104–106). IFN-γ secreted by activated CD8^+^ Trm cells enhances expression of vascular cell adhesion molecule-1 (VCAM-1) on vascular endothelium, which contributes to recruitment of CD4^+^ T cells, CD8^+^ T cells, and B cells to local tissues. In addition, TNF-α and IL-2Rβ-dependent cytokines are essential for DC maturation and granzyme B upregulation in both NK cells and bystander memory CD8^+^ T cells, respectively (105, 107). However, the crucial steps for the initiation of CD8^+^ Trm cell immune responses are still obscure. Further exploration should be focused on how to optimize their antiviral functions. A scheme for possible mechanisms of CD8^+^ Trm cells in viral protection is shown in Figure 3.

## Major open questions

It is now clear that CD8^+^ Trm cells play an important role in peripheral immune surveillance and protection against invading pathogens, especially in viral infection. The diversities of CD8^+^ Trm cells may be caused by different tissue microenvironments. The exact roles of different components involved in the process of CD8^+^ Trm cell mediated immunity are still obscure. Although great progress has been made in CD8^+^ Trm cell research, several problems need to be further explored: What is the origin of CD8^+^ T cell precursor in dLNs? What are the tissue-specific adaptations of CD8^+^ Trm cell development? What is the role of local tissue antigen-presenting cells in CD8^+^ Trm cell differentiation vs. recall reaction? How are CD8^+^ Trm cells regulated during reactivation? We believe that the strategies that modulate the functions of CD8^+^ Trm cells will be helpful for the control of viral infection if more details about CD8^+^ Trm cells are unraveled.

## Author contributions

XW and PW drafted the primary manuscript and figures. FX, YS, and XJ designed and corrected the manuscript. All the authors have read and approved the final manuscript.

### Conflict of interest statement

The authors declare that the research was conducted in the absence of any commercial or financial relationships that could be construed as a potential conflict of interest.
